# A policy roadmap for negative emissions using direct air capture

**DOI:** 10.1038/s41467-021-22347-1

**Published:** 2021-04-06

**Authors:** Jonas Meckling, Eric Biber

**Affiliations:** 1 Department of Environmental Science, Policy, and Management, University of California, Berkeley, CA USA; 2 School of Law, University of California, Berkeley, CA USA

**Keywords:** Climate-change policy, Political economy of energy

## Abstract

Negative emission strategies are central to avoiding catastrophic climate change. Engineered solutions such as direct air capture are far from cost-competitive. As past low-carbon technology transitions suggest, this calls for policy and political strategies beyond carbon pricing. We adopt a policy sequencing perspective that identifies policies that could create niche markets, building political support for later widespread deployment of direct air capture. Climate leaders could pursue an “incentives + mandates” policy strategy targeted at the oil and gas industry. These early moves could create global spillovers for follower countries by reducing technology cost and facilitating knowledge transfer through global firms.

## Abstract (web summary)

Negative emission technologies are central to avoiding catastrophic climate change. Deploying engineered solutions such as direct air capture requires a policy sequencing strategy that focuses on “incentives + mandates” in early adopters, while creating positive spillovers that incentivize follower countries to take policy action.

## Introduction

In the Paris Climate Accord, governments agreed to a goal of limiting total global temperature rise from climate change to 2 °C. Recent analyses by scientists have found that keeping warming to below 2 °C either requires extremely ambitious reductions of carbon emissions within the next decade, or extensive use of technologies and management techniques to remove carbon dioxide from the atmosphere. And achievement of a goal of limiting warming below 1.5 °C by the end of the century is now almost impossible without removal of carbon dioxide from the atmosphere^1–5^.

**Fig. 1:**
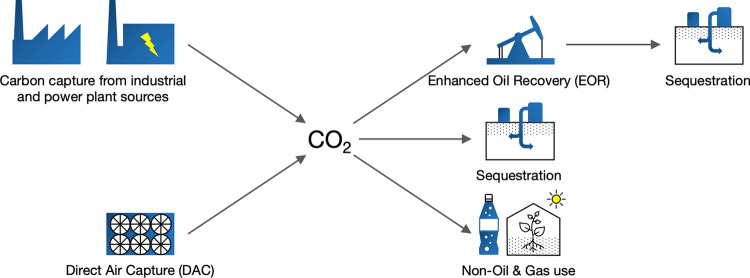
CO_2_ capture pathways. The figure depicts the two primary options for technological capture of CO_2_, and the primary use options for captured CO_2_, indicating which of the use options end up with permanent sequestration of the CO_2_. Sequestration refers to the injection of CO_2_ into deep subsurface rock formations for long-term storage. Non-oil and gas uses include chemical production processes, the beverage industry, and greenhouses.

A range of natural and technological approaches to remove carbon emissions from the atmosphere is available, with the approaches that have received the greatest analysis to date including reforestation and improved forest management; improved crop management; crops or forests used to produce energy, with the carbon emissions from the energy production being captured and sequestered in geological formations (bioenergy carbon capture and storage—BECCS); and direct air capture (DAC) of carbon dioxide from the atmosphere with the captured carbon being utilized or sequestered in geological formations (direct air capture and storage—DACS) (Fig. 1). Each of these approaches has limitations^1,3,4^. Reforestation and improved forest and crop management and BECCS all face land constraints, with related displacement of natural habitats and agriculture, and potential impacts on food prices. Carbon sequestration through reforestation and improved forest and crop management also faces concerns around permanency, as changes in land-use and impacts from climate change (such as increased forest fires) can release the above-ground carbon stored by these methods^3,4,6,7^. While these limitations do not foreclose the use of these other options, they do set upper limits on these approaches such that DAC will be an essential component in the full range of negative emissions technologies.

Thus, DAC has a significant appeal^8^. When combined with sequestration, it is a long-term and relatively permanent carbon removal technology that can address emissions from sources that are not easily or cheaply decarbonized^9^. While the land area required for the technology to capture the carbon is non-trivial, it is much less than that required for forest management or BECCS^1,3^. And while DAC has some infrastructure limitations (such as connection to energy sources and for some DAC technologies significant water sources, and for DACS to geological storage), it can be undertaken even in areas that are unsuitable for farming or forests. Despite its importance for climate policy this century, a market to advance DAC remains elusive, for two primary reasons.

First, DAC is currently very expensive, with cost estimates ranging from between $100 and $600 to potentially as high as $1000/ton of carbon removed^1,6^. A carbon price in that range would be significantly higher than the vast majority of carbon prices globally, many of which only cover a fraction of total greenhouse gas emissions^10–12^. Thus, while in theory, we could simply rely on setting the price of carbon “right” at a high enough level to transition to negative emission technologies over time, it seems politically implausible that we might achieve carbon prices at such a level in the timeframe required.

Second, unlike other lower-carbon technologies, such as renewable electricity and electric vehicles, DAC produces a primary product—CO_2_—for which there is currently limited market demand. That demand lies primarily in enhanced oil recovery (EOR), which refers to injecting CO_2_ into aging reservoirs to enhance oil production. EOR can only support a small fraction of the total amount of carbon that must ultimately be removed from the atmosphere. Similarly, the potential for demand growth in industrial carbon utilization beyond EOR is projected to be limited to 0.5% of the CO2 mitigation challenge by 2050^13^. Market development for DAC thus relies even more on policy support than other low-carbon technologies.

These challenges on the demand-side for DAC raise important political and policy questions that have so far been mostly unexamined^13,14^: What policies have the potential to effectively promote DAC deployment? Specifically, what policies that are politically feasible now have the potential to build future political support for the ambitious policy interventions we need to support DAC? And from an international perspective, which countries might move first, and how could they drive global deployment?

### Policies for climate leaders

The low-carbon technology transition is a problem of policy sequencing: initial policies need to bring down technology cost and broaden political support for the adoption of more ambitious policies for global technology diffusion^15–17^. Prior scholarship has emphasized the importance of research on which policies can and should accelerate DAC adoption^4,14^. Ongoing transitions to low-carbon technologies offer important lessons on what policy mix is most likely to drive investment and create a niche market that both can grow a new technology and support emerging interest groups that can advance additional policy. These suggest that the key policy formula is “financial incentives + deployment or performance mandates.” Financial incentives, such as subsidies or tax rebates have been pivotal to the deployment of renewable energy technologies and electric vehicles^18^. By 2014, 132 jurisdictions had adopted some form of incentive or mandate for renewable energy and 43 jurisdictions incentivized EV purchases. Such technology policies have been adopted much more widely than carbon prices. Given the cost of DAC, substantial government incentives will be necessary to make DAC viable. The US government introduced a tax credit (Section 45Q) for the geologic storage of CO_2_. This credit should be strengthened and other countries should follow suit in adopting financial incentives for DAC^19,20^.

Deployment mandates will also be critical for the uptake of carbon removal. Direct EV deployment mandates—as in China and California—and indirect deployment mandates through progressively stringent fuel economy standards—in, for example, the EU and the US—have been instrumental in the emergence of a nascent market for EVs. In renewables, some jurisdictions adopted deployment mandates, such as renewable portfolio standards, while others with very robust financing mechanisms—notably feed-in tariffs—relied solely on incentives. Relying solely on subsidies for DAC may ultimately become politically challenging as DAC scales up. The political reaction to the costs of feed-in tariffs for renewables as production increased in Germany and Spain provides an example of the risks, even with the revenue that the sale of electricity provides. The relatively lower political cost salience of mandates—compared to both taxes and subsidies—can allow for continued public support for the deployment of DAC. Thus, mandates are a crucial component for the effective scaling up of DAC. Oil and gas production or sales are the likely points of regulation, given existing revenue flows.

Mandating DAC can take various forms. The most important distinction is between upstream regulation (regulation of extraction of fossil fuels from the ground) and downstream regulation (regulation of the sale of fossil fuels for consumption by end users). Both options have potential and constraints. Upstream regulation could require that a certain amount of CO_2_ be captured and sequestered for every barrel of oil extracted. A key advantage of upstream regulation is that EOR provides an immediately viable economic use for DAC, which can facilitate initial investment and deployments. And the significant investments that oil and gas companies may have made in developing reserves can give jurisdictions significant leverage in imposing regulatory mandates. But upstream regulation is necessarily limited to jurisdictions that have substantial fossil fuel reserves to extract, which significantly constrains the number of possible jurisdictions that might adopt the regulation.

Even with the combustion of the oil extracted from EOR, EOR can produce net carbon sequestration when combined with anthropogenic sources of CO_2_, such as DAC^21–23^. Nonetheless, emissions from oil combustion, in addition to the limited amount of EOR capacity that is available^13^, mean that upstream regulation based on EOR cannot be the primary, long-term economic driver for most DAC or DACS needed to manage atmospheric carbon. EOR can, however, provide an initial policy jumpstart to create political support for the long-term driver of policies that can advance DAC or DACS. And while there is good evidence that EOR can provide permanent sequestration for much of the injected carbon dioxide^21^, additional research on leakage is required, as with all sequestration.

In terms of cost, while EOR can provide a revenue source for the carbon from DAC, that revenue may be partially or fully offset by changes to operations to increase the amount of carbon sequestered per unit of oil produced. For instance, revenue for DAC can range between $10 and 40/ton, depending on the price of oil. But using EOR techniques that maximize the storage of carbon dioxide can increase the cost of producing oil by over 50%^22^, while reducing the amount of oil produced per unit of CO_2_ injected and thus revenue. Thus, unless oil prices are high, policymakers using EOR to jumpstart DAC or DACS through upstream regulation will need to tradeoff between maximizing the amount of carbon sequestered through EOR and the cost required to support DAC or DACS^13^. Even with that tradeoff, however, the cost of a limited EOR program to build DAC or DACS programs need not be highly burdensome. The incremental cost for EOR that maximizes carbon storage to break even at an oil price of $50/bbl (incl. payment of $19/ton for CO_2_) is $30/Mt^22^. The EOR component need only be a relatively small part of overall global oil production—and therefore to have very minor impacts on oil prices—to provide a major driver of technological development for DAC and DACS. Using EOR to maximize carbon storage with 1.25 barrels of oil produced per ton of carbon dioxide sequestered^22^, and with global oil production of 90–100 million barrels/day, even relying on EOR for a few percent of total oil production could allow for large increases in the amount of DACS (currently at around 9000 tons/year with 1Mt/year in development)^24^, advancing technology and political economic support for policy.

**Fig. 2:**
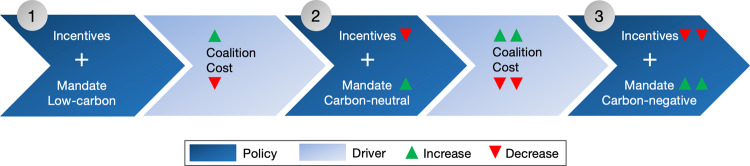
Policy sequence for climate leaders to develop DAC technology. Climate leaders, in particular those with an existing LCFS, could start market development for DAC through an “incentive + mandate” policy mix. Over time, they could ratchet up the stringency of mandates, while reducing incentives, as cost declines and political support broadens.

Downstream regulation can be applied across almost every jurisdiction in the world, given the ubiquity of fossil fuel consumption. For example, California’s Low Carbon Fuel Standard (LCFS), which mandates reductions in the carbon intensity of transport fuels, has included credits for oil produced from DAC since 2018. Despite having very high credit prices (as high as $180/ton in 2019^24^), the LCFS has been politically sustainable, indicating the leeway available to use such approaches to support DAC development. The federal government could reform the Renewable Fuels Standard along those lines. Also, states and provinces that adopted LCFS-style policies, such as Oregon and British Columbia, could follow California’s lead by including DAC. Globally, at least 70 jurisdictions had a biofuel and/or other transport fuel-related mandate in place by 2018 to which DAC requirements could be added^25^. In particular, the EU’s Renewable Energy Directive is offering an entry point for DAC mandates. Over time, policymakers could ratchet up fuel standards from low-carbon to carbon-neutral to carbon-negative (Fig. 2). While the oil and gas industry is the regulatory entry point for DAC mandates, a regulatory program could allow DAC credits to be generated by any firm. This could allow challengers from outside the oil and gas business that can develop innovative technologies and business models for the capture of CO_2_ and/or use of CO_2_ for long-lasting products to drive the DAC business. Tesla was instrumental in triggering technological change within the auto industry, while relying on credits of California’s LCFS as an important revenue stream in early days. In fact, the provision of credits to various groups, including electric utilities, automakers and oil and gas firms made the LCFS durable despite major pushback^26^. The “incentives + mandates” formula will be key for market development, but additional policies should include a public R&D program (e.g., US expansion of DAC R&D spending in FY 2020) and government procurement of DAC fuels^1,2,27^.

The history of low-carbon technology transitions suggests that creating government incentives for DAC will be the lower political hurdle, while mandating DAC presents a significantly greater challenge. But, as noted above, mandates can facilitate maintaining political support as DAC scales up. Moreover, mandates can be effective at creating markets for DAC. At least initially DAC will be a niche market with potentially lower profit margins for major oil and gas companies; mandates can force companies to make and continue DAC investments they might otherwise ignore or dismiss. Forcing this transition for incumbent oil and gas companies will be essential if carbon removal is not to extend the lifespan of oil and gas sales but instead is to develop a negative emission industry via lower-carbon and carbon-neutral fuels. Given the power of the oil and gas industry and its track record to delay the deployment of low-carbon technologies, significant political mobilization against oil and gas, including efforts to keep oil and gas in the ground^28^, will be needed to provide pressure on the oil and gas industry to pursue DAC. DAC mandates can be an integral part of such a mobilization. Accordingly, any subsidies for DAC should be politically tied to the progressive tightening of mandates for DAC deployment. An international alliance of frontrunner countries, a type of climate club^29^, could agree on increasing rates in low-carbon fuel regulation or, at a minimum, on review cycles for mandates. The “incentive + mandate” agenda is thus distinct from a pure subsidy agenda focused on EOR, and a real political litmus test for the oil and gas industry. Currently, oil majors are focusing their “decarbonization” strategies on natural gas, which is incompatible with the goals of the Paris Agreement.

The failure up to now of policy to advance carbon capture and sequestration (CCS) for power and industrial sources provides additional insights as to effective DAC policy. Governments have spent billions to attempt to advance CCS on fossil-fueled power plants and industrial sources but CCS has been deployed only at a limited number of sites around the world^30^. In theory, CCS should be lower cost because it can segregate carbon from emission flows with carbon dioxide concentrations much higher than the ambient air. But differences in the nature of standard CCS and DAC deployments to date can help explain in part why DAC policy may have more success than CCS has had. Deploying CCS on power or industrial sources requires large initial investments and long time-frames either to retrofit the equipment or to construct an entirely new source^31^. The primary mechanism to support CCS in the United States has been either rate-payer backed investments by incumbent utilities, or by state or federal government grants for individual projects. Because of the high cost, long time-frames, and large size of CCS projects, even substantial subsidies only permit construction of a limited number of projects^31^. This limits the number of entrants into the industry, as well as diminishing the benefit of repeated experimentation and failure that can facilitate learning-by-doing that can reduce technology costs^32^. The high cost of individual CCS facilities can also create political challenges for financing by creating a politically salient, expensive target for the opposition.

In addition, because of its deployment on new or existing power sources, CCS is geographically restricted to where those power sources are currently located, or to feasible sites for new power sources. Because CCS requires transportation of carbon dioxide to geological storage, it must be connected to individual pipelines or a network of pipelines unless the CCS facility is co-located with storage. Absent an existing pipeline system, such a system must be built from scratch, which can be costly, and the creation of such a network depends on overcoming significant network externalities, which can increase the challenges of developing CCS.

In contrast, DAC technology can be modular^33^, and to date most DAC facilities have been relatively small, decreasing the barriers to entry, increasing the opportunities for learning-by-doing, and reducing the political salience of individual projects. The granularity of DAC technology may also allow it to be ramped-up more quickly than CCS^33^. And because DAC location is more flexible geographically than CCS, it may be less dependent on transportation networks as DAC facilities can be co-located with geological storage, requiring only small-scale transportation systems^6^. Thus, DAC may be a more politically feasible approach for the development of carbon capture technology than CCS, which has been described as an “orphan technology^32^.” A key problem for all carbon capture technology is identifying a political economy framework that will create interest groups that will adopt an orphan—and indeed, the development of DAC may ultimately provide important policy and technological learning for CCS.

### The global leverage of leaders

**Fig. 3:**
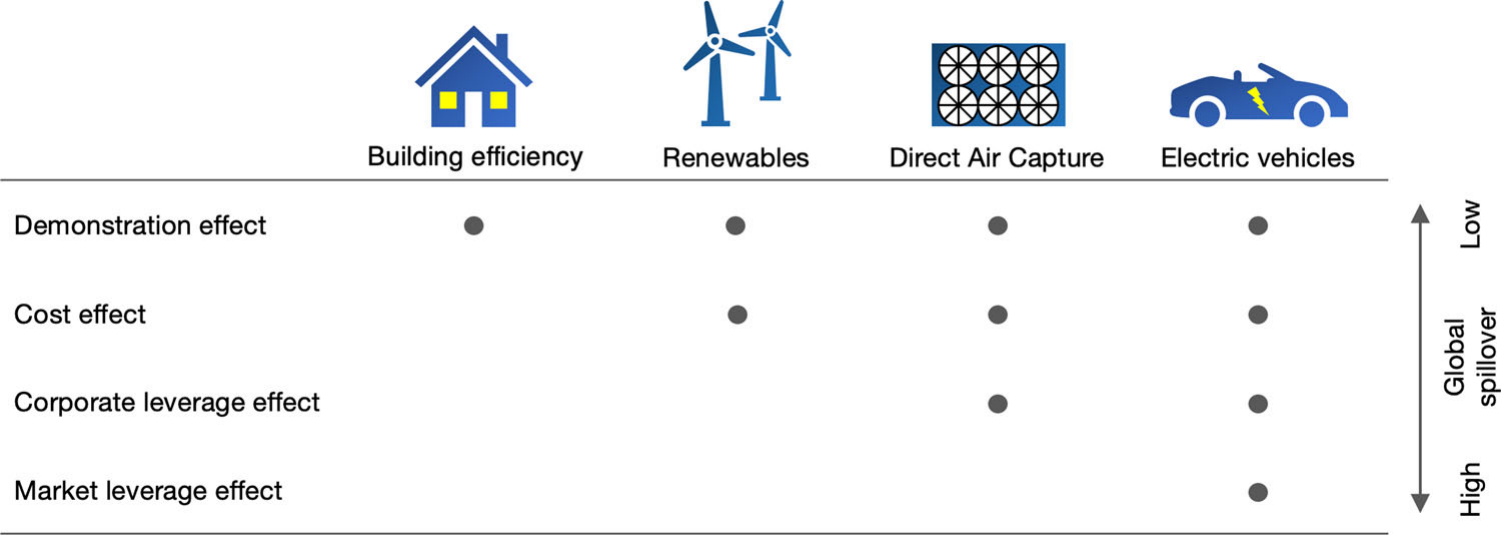
Global effects in technology transitions. The global leverage of climate leaders in technology transitions varies by how interdependent technology markets are. Depending on the level of interdependence, the spillovers of policy in a first-mover country to market actors in a follower country are more or less comprehensive. In all low-carbon transitions, climate leaders can create demonstration effects and often reduce the cost of technology for follower countries. In some cases, they can also influence follower markets through corporate leverage and market leverage effects.

In a global context, policy sequencing raises the question of how early adopters can influence later adopters and drive global technology transitions (Fig. 3). Emerging low-carbon transitions in the power and transport sector provide important lessons for the future of DAC.

#### Renewables and cost leverage

In many cases, early adopters with strong support policies in the form of subsidies and/or regulation can create niche markets that help bring down the cost of technology. Japan and Germany have done so in solar photovoltaics^34^. This reduces the cost of the transition for follower countries through economies of scale, research and development spillovers, network externalities, and path dependence^35^, while also creating a demonstration effect that the transition is doable. Beyond the cost and demonstration effects, the political effects of early renewables adoption have limited potential to spillover to other countries, given the nature of the electricity industry. Electric utilities are domestic companies operating in domestic or regional markets. This requires that renewable energy advocates build coalitions anew in every jurisdiction.

#### Electric vehicles and market leverage

Here again, policy leaders, such as California, Norway, and China drove early demand for electric vehicles, helping drive down technology cost^36^. Leaders had, however, important political effects beyond cost. Vehicle manufacturing is highly capital intensive, and automobile manufacturers generally develop and build vehicle models on a global scale, such that innovation in one market is reflected across all major markets. If a few large markets—such as California and China—change policy, they can shift the global product strategies of automakers, as happened with electric vehicles. This gives large lead markets in global industries outsized influence at a global level.

#### Direct air capture and corporate leverage

The development of DAC is likely to be both similar and different from the renewables and electric vehicle transitions. It will be similar in that large lead markets that invest in carbon removal technologies early on can help reduce the cost of the technology for late adopters. Especially low-complexity technologies, i.e., those with fewer components, are likely to experience more rapid cost declines than complex technologies^37^. DAC exhibit lower complexity than, for example, electric vehicles, suggesting a steeper learning curve.

The transition to carbon removal technologies will likely differ from the solar and EV transitions in its politics. The structure of the oil and gas industry—a sector central to the development of DAC—differs from the utility and auto sectors. Oil and gas firms possess the capital, the global infrastructure, and the knowledge to develop a process like DAC that requires the knowledge of chemical and mechanical processes. Most importantly, however, they have demand for CO_2_ and a product—oil and gas—that offers a regulatory entry point for DAC and DACS deployment. Oil and gas firms have long employed EOR to enhance oil and gas production; they have also built pure geologic CO_2_ storage outside of EOR. However, lead markets on DAC are unlikely to shift the strategies of oil majors across all markets, as happened in the case of electric vehicles. DAC and DACS technology would be separate and stand-alone from core oil and gas extraction technologies. Accordingly, oil majors need likely only deploy DAC and DACS technology where it is required, making it divisible across markets such that unilateral regulation by one large-market jurisdiction cannot force deployment globally, unlike EVs. However, the global nature of oil and gas firms lends itself to knowledge transfer across markets. Once political pressure mounts to mandate carbon removal in follower markets, oil firms will be well-equipped to rapidly scale up DAC and DACS deployment. Such “corporate leverage” differs from the renewables transition, where utilities in new markets need to learn to transition their business model from scratch.

### Lessons for next steps

The global sequencing perspective on developing markets for DAC technologies suggests two key lessons. First, a set of lead markets need to start mandating the progressive deployment of DAC to drive investments by the oil and gas industry and other players, while providing deployment incentives. For downstream regulation, likely candidates are jurisdictions with high public demand for climate action and no significant domestic oil and gas industry. These include in particular continental European countries, such as Denmark, France, Germany, the Netherlands, and Sweden, but also US states, such as New York and Massachusetts. A second group of potential lead markets includes jurisdictions with public opinion in favor of climate regulation and some oil and gas industry, such as California, Norway, China, Canada, and possibly Middle Eastern countries, such as Qatar which could implement upstream and downstream regulation^38^. These countries could form a global alliance for the deployment of carbon removal technologies, including DAC and related technologies. Similar technology-related leadership groups have emerged on solar—the International Solar Initiative, on electric vehicles—the Electric Vehicles Initiative, and on coal power moratoria—the Powering Past Coal Alliance.

Second, the global diffusion of DAC cannot rely on strong market leverage effects like the transition to electric vehicles. It will require a sustained climate mobilization in favor of DAC regulation across countries akin to the diffusion of renewable energy policies. Oil and gas firms can easily choose to deploy DAC in some markets but not in others. This also raises the possibility of the creation of new companies focused on DAC only. They might play an important role in supporting the development of DAC policies—as project developers have done in renewable energy—and provoke action on the part of incumbent firms—as Tesla has for EVs. At the same time, the corporate leverage effect suggests that targeting oil and gas firms—through public advocacy and investor activism—can potentially facilitate cross-country technology transfer given the firms’ global scale.

Over the past few years, the climate policy community has come to realize the inevitable need for negative emission technologies. It is time to move from policy dreams of high carbon prices to tested and pragmatic strategies of sectoral technology transitions.
